# Tracing the Bioavailability of Three-Dimensional Graphene Foam in Biological Tissues

**DOI:** 10.3390/ma10040336

**Published:** 2017-03-24

**Authors:** Tanveer A. Tabish, Sakineh Chabi, Muhammad Ali, Yongde Xia, Farhat Jabeen, Shaowei Zhang

**Affiliations:** 1 College of Engineering, Mathematics and Physical Sciences, University of Exeter, Exeter EX4 4QF, UK; tt302@exeter.ac.uk (T.A.T.); schabi@fit.edu (S.C.); Y.Xia@exeter.ac.uk (Y.X.); 2 Department of Chemistry, Florida Institute of Technology, 150 W University Blvd, Melbourne, FL 3290, USA; 3 Department of Zoology, Government College University, Faisalabad 3800, Pakistan; alisam0017@gmail.com (M.A.); farhatjabeen@gcuf.edu.pk (F.J.)

**Keywords:** graphene foam, bioavailability, biological tissues, fish

## Abstract

Graphene-based materials with a three-dimensional (3D) framework have been investigated for a variety of biomedical applications because of their 3D morphology, excellent physiochemical properties, volume stability, and their controllable degradation rate. Current knowledge on the toxicological implications and bioavailability of graphene foam (GF) has major uncertainties surrounding the fate and behavior of GF in exposed environments. Bioavailability, uptake, and partitioning could have potential effects on the behavior of GF in living organisms, which has not yet been investigated. Here, we report a pilot toxicology study on 3D GF in common carps. Our results showed that GF did not show any noticeable toxicity in common carps, and the antioxidant enzymatic activities, biochemical and blood parameters persisted within the standard series. Further histological imaging revealed that GF remained within liver and kidney macrophages for 7 days without showing obvious toxicity. An in vivo study also demonstrated a direct interaction between GF and biological systems, verifying its eco-friendly nature and high biocompatibility.

## 1. Introduction

Recent development of three-dimensional graphene foams (3D GF) [1] provides an effective route to uniform dispersion of graphene in a composite matrix [2,3]. The effective and homogeneous distribution of graphene has been the focus of substantial investigations, with the most critical changes in morphology and porous architecture [4]. 3D GFs form a united and continuous network of graphene sheets, thus fulfilling the requirement of uniform distribution [5]. 3D GFs could be potentially used in a variety of areas, such as in energy storage [6], Li ion batteries [7], supercapacitors [8], electrochemical sensing [9], and tissue engineering (as stem cell scaffolds) [10] owing to their high surface area (ranging from a few hundred to ca. 2000 m^2^/g) and hierarchical (macro/meso/micro pores) structure in combination with the intrinsic properties of two dimensional (2D) graphene. 3D GFs are economical to produce and highly scalable for commercial and industrial applications [11]. Recently, the biocompatibility of GF in living systems has become a great concern [11], although it shows great potential in stem cells and some other applications. Regardless of these applications, studies on the direct biological interaction of GF with living and aquatic system are not available. So far, only a few studies have exploited GF’s porous morphology and architecture for neural and human mesenchymal stem cells, bioactive scaffolds, and drug delivery system. Wang et al. [12] used polycaprolactone-enriched GF (PCL/GF) as a promising scaffold for bone tissue engineering because of its excellent biomineralization rate and the presence of a hydroxyl group. Nieto et al. [13] fabricated a high strength biocompatible scaffold via forming a thin uniform PLC coating on GF using a dipping method. Although these reports demonstrated in vitro applicability of GF in tissue engineering, the bioavailability and potential toxic effects of 3D GF in living models remains unclear. Assessing the potential impacts of GF on the human health is critical for the sustainable development of the graphene-industry. Bioavailability and uptake of GF to organisms are key determinants of toxicity, yet these features are useful and coherent modes of aquatic animals. This work addresses this omission by focusing on these important principles for GF.

In the present study, GFs were probed as an in vivo material for clinical trials. Common carps (*Cyprinus carpio*) are a remarkable class of species in freshwater environments and are commonly used as an in vivo model. Common carp is fundamentally an important aquatic species for toxicology of nanomaterials (NMs) [14]. Compared to laboratory fishes, common carps are stronger against contaminants mainly due to the variety of their interaction routes, multiple exposure routes into organisms, physicochemical characteristics of water, and diversity of aquatic environment [15]. These are generally considered the most appropriate model to evaluate the properties of toxins and their implications on a biological system. GFs were shown to maintain remarkable biocompatibility, low responsiveness to toxicity screening, and very small fluctuations in enzymatic patterns of common carp [15]. In the present work, we investigated the interactions of GF with fish, representing antioxidant enzymatic activities (superoxide dismutase (SOD), catalase (CAT), and glutathione-S-transferase (GST) in vital tissues such as the liver, kidney, and heart), biochemical features in the blood and histological alterations in the liver, kidney, and heart, and also assessed their bioavailability to fish (common carp) that were exposed for 7 days. These findings would help to explore and develop novel and facile GF-based approaches for tissue engineering and regenerative medicine.

## 2. Results and Discussion

**Figure 1:**
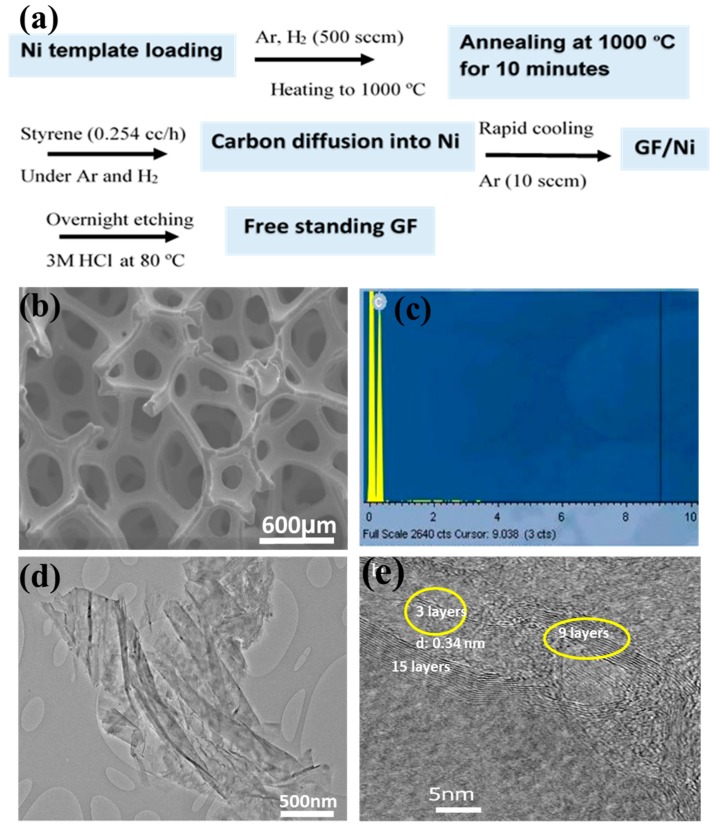
(**a**) A schematic showing the typical graphene foam (GF) formation process; (**b**) Scanning electron microscopy (SEM) image; (**c**) Energy dispersive X-ray spectrometry (EDS); and (**d**,**e**) Transmission electron microscopy (TEM) images, of GF.

GFs were fabricated via chemical vapor deposition (CVD) of graphene on a Ni foam template, as schematically illustrated in Figure 1a. The Ni scaffold assisted CVD process is an effective way to obtain larger grains for better quality growth and to produce GF with a controlled morphology. The porosity, grain size, and surface smoothness of three-dimensional (3D) Ni foam with visible grain boundaries make it suitable for GF growth. As-prepared GFs were characterized by transmission electron microscopy (TEM), scanning electron microscopy (SEM), and energy dispersive X-ray spectrometry (EDS). Figure 1b–e present together SEM, EDS, and TEM images of an as-prepared GF. EDS (Figure 1c) shows only one carbon peak at 0.4 KeV, but no Ni peaks, indicating the successful removal of the original Ni template after the acid leaching. SEM (Figure 1b) reveals that the as-prepared GF had a porous interconnected 3D network. The TEM (Figure 1d,e) further reveals that they are comprised of multi-layered graphene. Figure 1d shows a “large” sized (about 4 µm) graphene sheet with folded areas. Figure 1e gives an HRTEM image of graphene sheets, revealing that the layer number varied from 2–3 in some sheets to 9–15 in others.

As is well known, it is very challenging to control the number of graphene layers, with the Ni-assisted CVD method. The interlayer spacing was found to be 0.34 nm which matched with the d value calculated from the XRD data.

**Figure 2:**
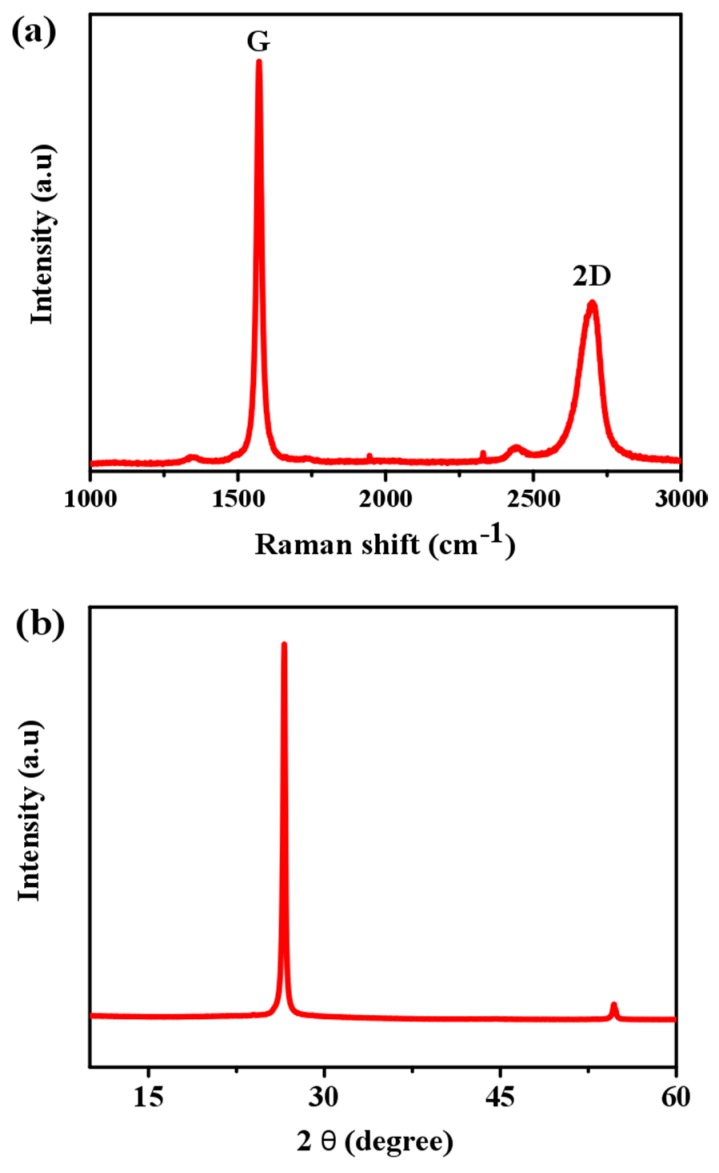
(**a**) Raman spectroscopy and (**b**) XRD of GF with Cu-Ka radiation (l ¼ 0.154 nm) operated at 40 kV and 40 mA.

GFs were also characterized by Raman spectroscopy. As seen in Figure 2a, two strong peaks at 1574 cm^−1^ (G) and at 2720 cm^−1^ (2D) appeared. The position and intensity of the Raman peaks give valuable information about the defect level, the number of graphene layers or the sp^3^ hybrid phase. The G peak is the E2g optical mode of graphite and this band arises from the C=C in-plane stretching vibration. Negligible effect of the D mode at ≈1300 cm^−1^ indicates a perfect crystal structure of the foam, and a carbon monolithic-like structure [16,17,18,19]. As shown, the G peak (intensity: I_G_) is stronger than the 2D peak (intensity: I_2D_), suggesting the few layer feature of the GF (I_G_/I_2D_ ~ 2.4). In a single layered graphene, I_2D_ is greater than I_G_, whereas in a bilayered graphene, both are almost equal. Figure 2b shows the XRD pattern of a powder sample prepared from as-prepared GF. The sharp peak at 26.5° (2θ) corresponds to the (002) plane of graphite, and the weak one at ca. 55° to the (004) plane.

As-prepared GFs were further investigated for toxicity and spontaneous morphological and histological changes in common carp. Potential toxic effects, bio distribution of NMs and their uptake, and identification of target organs in carp fish were assessed in a variety of factors as elucidated in Figure S1.

In the present work, common carps were investigated for 7 days following treatments with low (5 mg/L), medium (10 mg/L) and high (15 mg/L) doses of GF. Animal models were distributed in four groups, three treated with low, medium, and high doses and one untreated as a control group, *n* = 8 per group. The control and treated animals were then euthanized for histological analysis, biochemical parameters, enzymatic activities, and further studies. There were some additional animal models involved in this experiment, as a safe side of the experiment. Body weights were supervised and measured every 48 h and the variations were very close between treated and untreated groups (Figure S2), suggesting insignificant systemic effects. The eating, drinking, experimental conduct, grooming, urination, and neural changes were normal throughout the 30 day study. No nausea was detected before and after the treatment of GF formulation.

**Figure 3:**
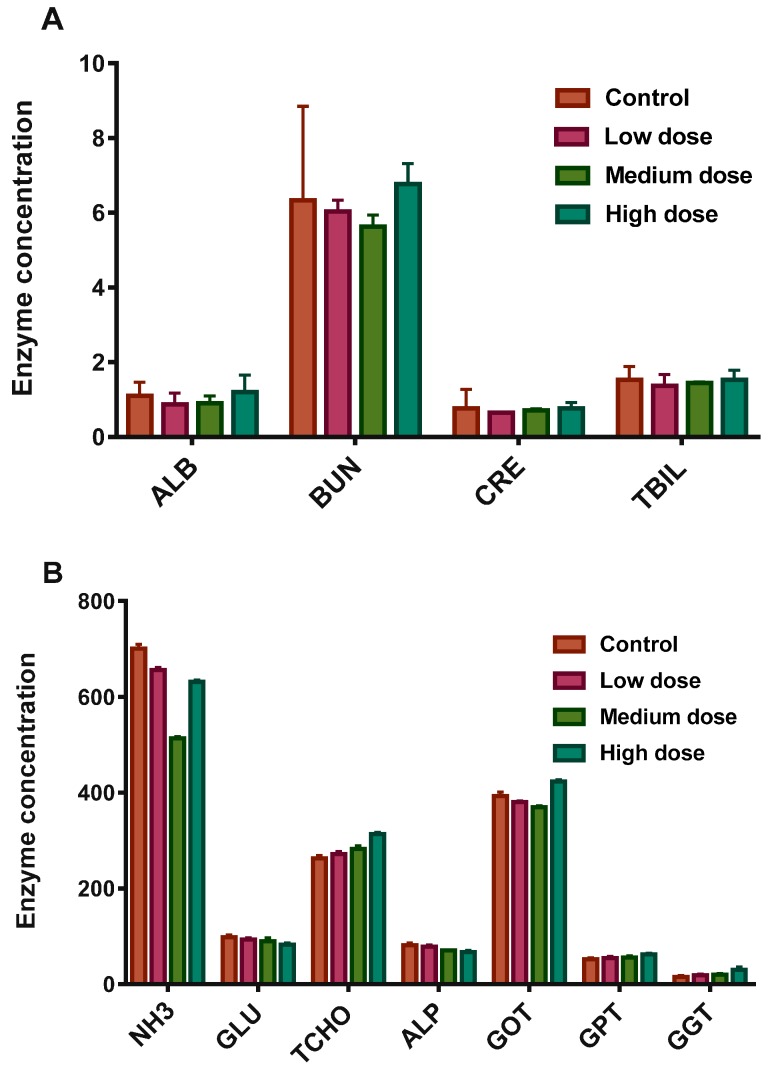
(**A**,**B**): Blood analysis of common carp exposed to GF as a function of dose level after 7 days. These results showed mean and standard deviations of ALB: albumin (d/dL), BUN: blood urea nitrogen (mg/dL), CRE: creatinine (mg/dL), TBIL: total bilirubin (mg/dL) and B: NH3: ammonia (μg/dL), GLU: glucose (mg/dL), TCHO: total cholesterol (mg/dL), ALP: alkaline phosphates (μ/L), GOT/AST: glutamic oxaloacetic transaminase/Aspartate Aminotranferse (μ/L), GPT/ALT: alanine aminotransferase (μ/L), GGT: ν-glutamyltransferase (μ/L), ALB: albumin (d/dL), BUN: blood urea nitrogen (mg/dL), CRE: creatinine (mg/dL), and TBIL: total bilirubin (mg/dL). Data are presented as mean ± SE (*n* = 3).

The biochemical parameters of treated and control fishes were examined for any acute and appreciable marks of toxicity and their responses to GFs over 7 days. These features did not show any significant changes. Figure 3A,B presents blood testing results of common carps exposed to GF in a dose-dependent manner after 7 days, including NH_3_: ammonia (μg/dL), GLU: glucose (mg/dL), TCHO: total cholesterol (mg/dL), ALP: alkaline phosphates (μ/L), GOT/AST: glutamic oxaloacetic transaminase/Aspartate Aminotransferase (μ/L), GPT/ALT: alanine aminotransferase (μ/L), GGT: ν-glutamyltransferase (μ/L), ALB: albumin (d/dL), BUN: blood urea nitrogen (mg/dL), CRE: creatinine (mg/dL), and TBIL: total bilirubin (mg/dL). Data represent the average ± SD (*n* = 3). No statistically significant changes were observed between different groups in a dose dependent manner except the BUN. The assessment of the biochemical parameters revealed that a higher dose of GF likely had a toxic effect because of its strong hydrophobic interface with cell membranes [20], although GF exhibited an insignificant hemolytic effect (up to 75 μg/mL) and minor intensities of coagulation. However, graphene oxide (GO) and their other counterparts at 2 μg/mL provoked persistent and severe injury in lungs [20]. GF did not induce appreciable toxic effects in serum biochemical levels because of its different morphology, chemical structure, higher surface area, and porous architecture as compared to other graphene-based counterparts.

Next, we studied antioxidant enzyme activities before carrying out the histological analysis on the vital organs. It should be noted that the antioxidant enzyme expressions and levels are accountable for the removal of chemically induced oxidative stresses in the immune and defensive mechanism of a living system. Antioxidants include several enzyme classes such as Glutathione-S-transferase (GST), catalase (CAT), and superoxide dismutase (SOD). Irregular abnormalities in these enzymatic repairs reveal the level of oxidative damages and defense. Variations in oxidative lesions have also recently been found to be a main factor for tumor growth in the liver as a result of polluted environment [21]. Exposure of nanoparticles (NPs) induces the mitochondria damage (via) depletion of glutathione, an endogenous thiol (SH–) group, and stress proteins. These antioxidants and free radicals are mediators of tissue and cellular related injuries and diseases [22,23]. Also the increased bioaccumulation of NPs causes a steady rise of hepatic and renal antioxidant activities, affecting the mitochondrial respirational system [24]. Hence, there must be a balance between generation of oxidants and antioxidants and the level of lipid peroxidation in vital tissues of the carps. Environmental stress is also involved in the functions of aquatic organisms.

**Figure 4:**
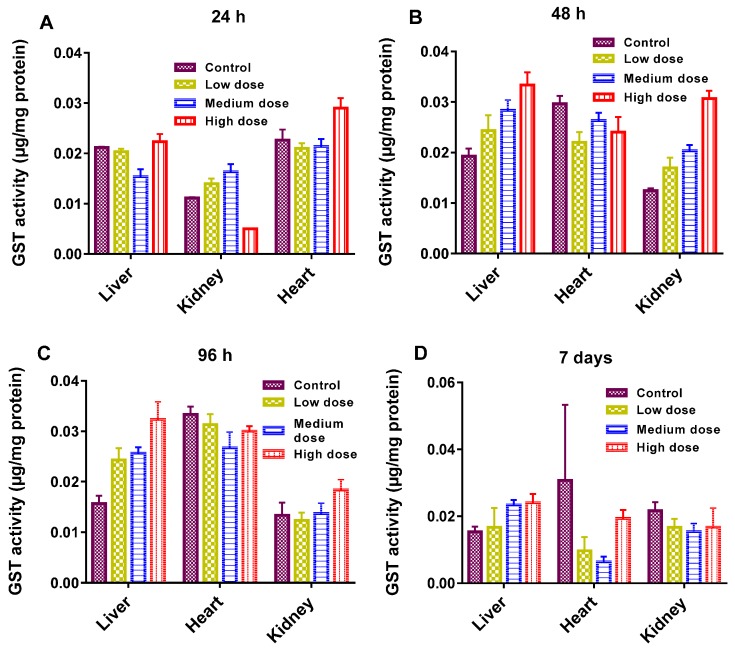
(**A**–**D**): Glutathione-S-transferase (GST) activity in different organs of the common carp exposed to various concentrations of GF for different times: (**A**) 24 h (**B**) 48 h (**C**) 96 h (**D**) 7 days. Data are presented as mean ± SE (*n* = 3).

**Figure 5:**
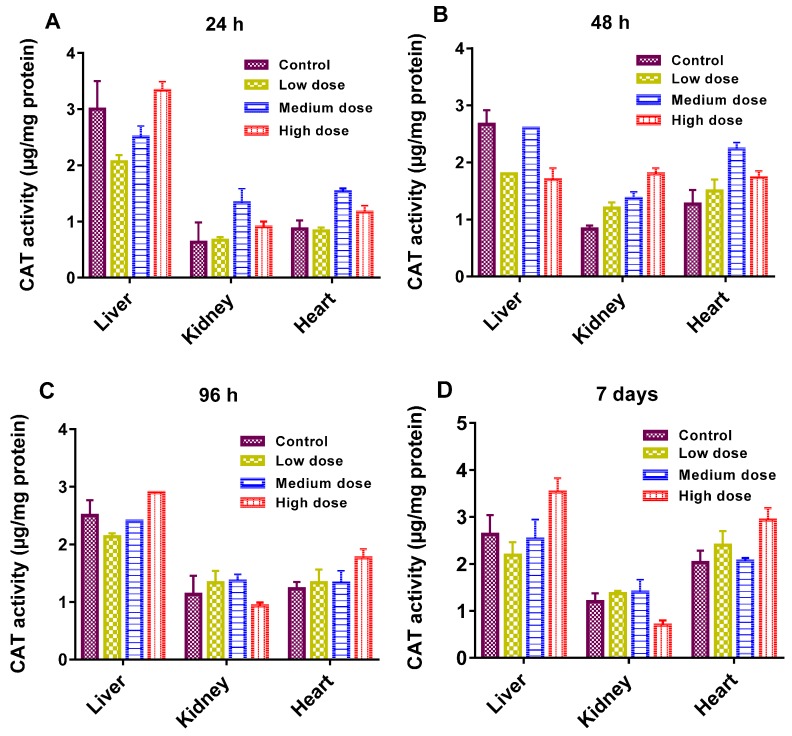
(**A**–**D**): catalase (CAT), activity in different organs of the common carp exposed to various concentrations of GF for different times: (**A**) 24 h (**B**) 48 h (**C**) 96 h (**D**) 7 days. Data are presented as mean ± SE (*n* = 3).

**Figure 6:**
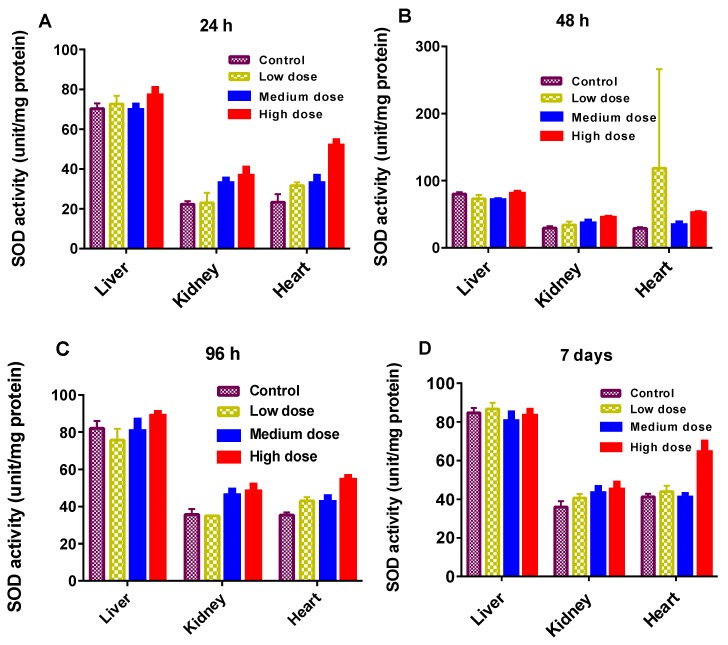
(**A**–**D**): Superoxide dismutase (SOD) activity in different organs of the common carp exposed to various concentrations of GF for different times: (**A**) 24 h (**B**) 48 h (**C**) 96 h (**D**) 7 days. Data are presented as mean ± SE (*n* = 3).

The antioxidant enzymatic activities (GST, CAT, and SOD) are presented in Figure 4, Figure 5 and Figure 6. As shown, enzymatic activities generally showed variation in a dose-dependent manner. GST actions in the liver, kidney and heart were normal regardless of the GF exposure after 48 h (Figure 4A). While at a higher dose, GST declined more significantly in the kidney than in the liver and heart and at 96 h, and it was prominent in the liver at a higher dose (Figure 4B). GST plays a catalytic role in conjugation of toxic and harmful metabolites. Higher levels of GST cause the activation of enzymes involved in glutathione (GSH) synthesis. GSH indicates amplified detoxification activities in the main organs of fish [25]. The decline in GST levels was an effect of the overuse of enzymes to resist the oxidative stresses instigated by GF, eventually GSH concentration was increased in vital tissues. In this work, the common carp revealed a substantial increase in GST over a 24 h and a 7 day timescale, but oxidative stresses decreased with increasing the GST concentration. Therefore, a rise in GST concentration can also be used for the analysis of reduced GSH dependent metabolism changes involved in redox and detoxification processes.

The key role of CAT is to protect the cells from accumulations of H_2_O_2_ by catalyzing its decomposition to H_2_O and O_2_, and to activate H_2_O_2_ as a peroxidase [26]. Its levels were similar among the control and treated groups, except a slight change in the kidney tissues of the common carp at a 7 day timescale indicated a reduced activity to protect the cells against H_2_O_2_ (Figure 5). It was reported that the enhanced SOD and CAT in the hepatocytes of the fish might be prompted by microcystin [27].

SOD levels were within the range observed for control and treated carps (Figure 6). SOD is a defensive free radical in enzyme systems that principally dismutase superoxide radicals [28]. This also reveals the greater requirement of proteins to protect the cells against the radicals. However, SOD activity was considerably lesser in the liver of fish exposed to high dose as compared to the liver in the control model (Figure 6A). The antioxidant resistance of the liver was affected at higher concentration, as evidenced by CAT and GST in the liver of carp exposed to a low dose. Based on these results, we consider that GST and CAT were generated in appropriate capacities to neutralize the oxidative stress produced by GF. However, the relationships between GST, CAT, SOD and other antioxidant enzymes need to be established by further investigations.

**Figure 7:**
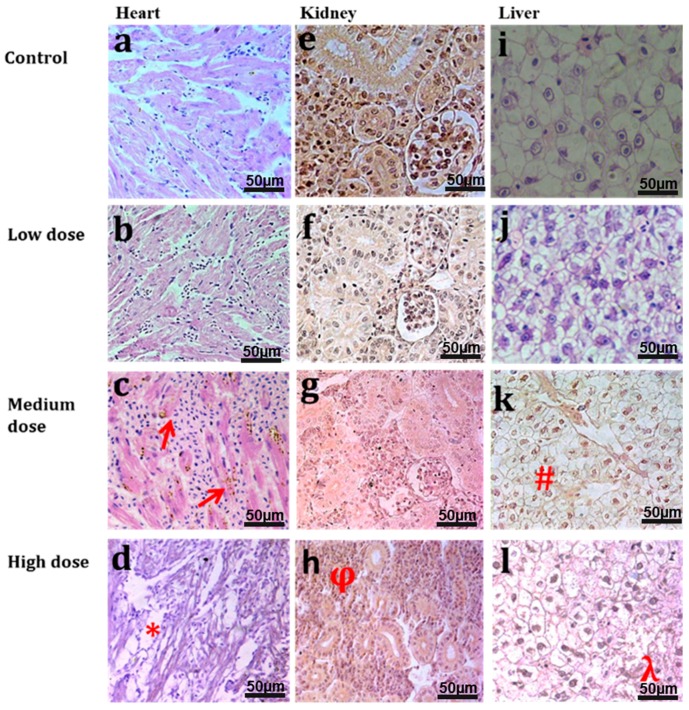
H & E stained light micrographs of *Cyprinus* *carpio* {heart (**a**–**d**), kidney (**e**–**h**) and liver tissues (**i**–**l**)} treated with GF in a dose dependent manner. Figure 7a,b show normal histology of heart tissues, Figure 7c,d show histological alterations with deposition of lipofuscin granules (**c**) and degeneration/thinning of cardiac muscles (**d**) in high dose. Figure 7e,f show normal histology of kidney in the control and low dose treated groups while atrophy and constriction of glomerulus was found in the medium treated group (**g**). Necrosis and degeneration (ϕ) of kidney tubules was found in the high dose treated group (**h**). Normal histology of fish liver was found in the control and low dose treated groups (**i**,**j**) while degeneration of hepatocytes (#), pyknosis, karyolysis and karyorrhexis in nuclei of hepatocytes (**k**,**l**) and degeneration of central vein in liver lobule of *Cyprinus carpio* (**k**) were found in the medium and high dose treated groups.

Histopathology of the heart, kidney, and liver of the common carp was also exploited and the results are shown in Figure 7 (in a dose dependent manner). Less damage was revealed in the low dose groups, but more damage was perceived in the high dose groups. Heart tissues showed normal histology in the control and low dose treated common carps (Figure 7a,b), whereas brown atrophy (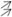) was found in the fish heart treated with medium dose (Figure 7c) due to the deposition of pale golden brown (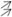) (lipofuscin) granules in the heart muscle fibers. Common carp treated with a high dose showed degeneration of muscle fibers (*), vacuolization and thin fibers (Figure 7d). Figure 7e–h show micrographs of the kidney of common carp treated with different doses of GF. Normal histology of the kidney was observed in the control and low dose treated groups (Figure 7e,f). Atrophy and degeneration of glomerulus was found in the medium treated group (Figure 7g). Necrosis and degeneration (ϕ) of kidney tubules was found in the high dose treated group (Figure 7h). Normal histology of the fish liver was found in the control and low dose treated groups (Figure 7i,j) while degeneration of hepatocytes (#), pyknosis, karyolysis, and karyorrhexis in nuclei of hepatocytes and degeneration of the central vein in the liver lobule of common carp were found in the medium dose treated groups. High levels of hepatocytes degeneration (λ), karyorrhexis, and haemorrhage were also found in liver lobule of fish treated with a high dose of GF (l). The respective histopathological alterations in these vital tissues of both control and treated groups (Figure 7) are given in Table S1. Histological alterations in these organs after 5 days of GF treatment are also shown in Figure S3 and Table S2.

No noticeable toxicity was found after breakdown of GF in vivo over a timescale of 7 days. However, the biodistribution and toxicokinetics in the non-human primates (NHPs) investigation revealed remarkably biocompatible with GF, which could offer a new avenue for future toxicology studies of GF. Common carp treated with a high dose of GF survived without any sign of toxicity. The enzymatic and anti-oxidant activities were used to define the ultimate fate of GF in living systems. All results from this work suggested that as-prepared GF had excellent biocompatibility on the timescale investigated. These could unveil the potential risk associated with their bioaccumulation. The relative infancy of NMs begs for animal model investigations to shed light on in vivo interactions of NMs before translation to humans. These findings also raise an interesting concern as to what level do histopathological variations of exposed GF in the common carp model indicate toxicity in primates? It is necessary to use appropriate models to assess and optimize the fundamental mechanism of NPs toxicity and compatibility before clinical applicability. Although these methodological studies indicated the toxic effects in such animal models and were useful for research, it is really hard to relate these responses and effects to those in humans. A cross-species comparative approach can significantly improve the prediction of human responses to practical and realistic applications. These investigations usually lay the groundwork for clinical applicability, based upon the evident physiological and functional resemblances amongst NHPs and humans.

A large number of in vivo studies based on histology changes of vital organs exposed to graphene have been carried out before. A non-biodegradable feature of GFs as implanted scaffolds was demonstrated in rat exhibiting good biocompatibility [29]. GO administration in some other animal models, such as rat, caused prolonged toxicity and lung granuloma death [30]. In another study, GO administration was found to induce dose-dependent lung toxicity, granulomatous abrasions and injuries, and inflammatory cell penetration [31]. Higher concentrations of graphene, GO and rGO (reduced graphene oxide) were reported to be toxic. Fortunately, the results from the present study indicated that toxicity of GF was very minor. This is probably because of its different porous structure, chemical and physical morphology, and architecture, compared to those of its other graphene-based counterparts. Synthesis routes, size, surface charge, colloidal stability, surface chemistry, and water solubility affect in vivo nano-formulations. No noticeable differences were found in the in vivo toxicity of GF in this study. Additionally, GF appeared to be non-biodegradable even after 7 days of treatment. Several factors might have contributed to the toxicity mechanism of GF, including the variety of exposure routes to living models, short or long term exposure periods, different chemiophysical properties, volume stability, and surface properties in vivo. To solve the real world clinical problems, these factors must be considered before evaluation of toxicology and bio-distribution of NPs. Hence, understanding the fundamentals of aquatic toxicology and bioavailability of GF would also provide insights into the validity of environmental fate and impacts of GF. Long term toxicological and biodegradability studies of GF rooted into the target tissue for regenerative engineering need to be carried out in the future.

## 3. Materials and Methods

### 3.1. Fabrication and Characterization of Three-Dimensional GF

Graphene foams were prepared via a CVD route using styrene and a Ni foam template (supplied by Novamet, USA, with a 99% porosity and 1.6 mm thickness). Briefly, the Ni template was activated in a tube furnace at 1000 °C for 10 min under Ar flow of 180 mL/min and H_2_ flow of 200 mL/min, followed by injecting the styrene carbon source into the furnace tube at a rate of 0.254 mL/h (controlled by a syringe pump (Razel Scientific Instrument, Inc., USA)), still under the same mixture gas flow for 1 h. Finally, the sample was cooled down naturally to room temperature under a reduced Ar flow of 50 mL/min. The 3D graphene networks were obtained by overnight etching of the original Ni template in 3 M HCl, and the final product was characterized by using a scanning electronic microscope (SEM) (Hitachi S3200N SEM-EDS, Japan). X-Ray diffraction analysis was performed on a Bruker D8 Advance diffractometer with Cu-Ka radiation (l ¼ 0.154 nm) operated at 40 kV and 40 mA. Raman spectra were recorded on a Renishaw Raman microscope. The excitation laser beam at a wavelength of 532 nm was focused by a 50× objective onto a small area of the sample. A transmission electron microscope TEM (JEM-2100 TEM) was also used to characterize the sample. For TEM examination, GF samples were ultrasonically dispersed in acetone (Fisher Chemical, UK) for 30 min, and then the suspension was dropped onto a holey carbon coated copper grid (300 mesh, Agar). This procedure was adopted from our previously published work [32,33].

### 3.2. Procedure for In Vivo Toxicity

Common carp (*Cyprinus carpio*) (50 ± 2 g weight and 29 ± 0.9 cm in length) was procured from the Fish Hatchery Satiana Road Faisalabad Punjab, Pakistan and held there for two weeks in a stock aquarium with flowing aerated dechlorinated tap water. Stock fishes were fed with commercial fish meal, and maintained in the stock aquarium at 28 ± 2 °C and 12:12 light to dark period (after permission by the ethical committee of Government College University, Faisalabad, Pakistan). After 2 weeks acclimatization, fishes weighing around 50 g (*n* = 40) were transferred into four aerated experimental glass aquaria (10 fishes/tank) and further acclimated for 48 h. They were randomly divided into four groups having the non-significant difference in weight. The first group was used as the control group (without GF treatment), and the other groups were exposed to either 5 (low dose), 10 (medium dose) or 15 (high dose) mg·L^−1^ of sterile GF for 7 days. During the test period, the fishes were fed twice a day with artificial diet. Both blood and tissues (heart, kidney and liver) were collected after 24, 48, 96 h and 7 days of exposure for each treatment, randomly. Blood samples were collected through cardiac puncture by using 2 mL heparinized needle flushed with EDTA and transferred to a tube containing EDTA. The tissues were frozen at −4°C for further analysis. For histological analysis, heart, kidney and liver tissues with a diameter of 3–5 mm were fixed in sera (60% ethanol + 30% formalin + 10% acetic acid) for 3–4 h [34]. The fixed samples were dehydrated at room temperature with ethanol and toluene series and embedded in paraffin. These paraffin embedded tissues were sectioned into thin slices of 4–5 μm by using a microtome (SLEE Rotary Microtome CUT5062 by Nikon Instruments Europe), stretched in water and mounted on gelatin-coated marked glass slides. These sections were then stained with haematoxylin and eosin. The stained tissues were examined under a light microscope (Nikon Eclipse 50i by Nikon Instruments Europe) fitted with a digital camera.

### 3.3. Measurement of Enzymatic Activity and Other Biochemical Parameters

Liver, heart and kidney samples from the fishes were collected at different timescales after treatment, ice-covered, and kept separately at ~20 °C. These sections were washed with 0.15 mM KCl solution and normalized on ice with 50 mM phosphate buffer (pH 7.0). The suspension was sonicated and then centrifuged (at rate of 10,000× *g* at 4 °C for 10 min). GST activity was measured using a GST Tag assay kit (Novagen, Germany). The reaction absorbance was monitored at 340 nm by using a UV spectrophotometer (Tecan Infinite F200, Austria). CAT activity was measured using the Abei method [35]. SOD activity was measured by using an SOD assay kit (Dojindo Laboratories, Kumamoto, Japan). Total protein concentration was calculated by using the Bradford method [36]. The biochemical parameters (total cholesterol (TCHO), alanine aminotransferase (GPT/ALT), albumin (ALB), alkaline phosphates (ALP), ammonia (NH_3_), glucose (GLU), ν-glutamyltransferase (GGT), glutamic oxaloacetic transaminase/aspartate aminotransferase (GOT/AST), blood urea nitrogen (BUN), creatinine (CRE), and total bilirubin (TBIL)) were examined in the current work. Statistical records were measured and analyzed using Excel software and plotted using the origin pro 2016 version. The differences between the samples and controls were assessed using one-way Anova. Data are presented as mean ± SE (*n* = 3).

## 4. Conclusions

The present work deals with the systematic toxicity assessment of GF in common carp. High dose administrations did not clue to critical or prolonged toxicity in fish, but some variations in blood cells were observed. In terms of biochemical and blood parameters testing, values remained within standard series resulting in no morphological and metabolism changes in fish model. Histopathology imaging revealed that GF remained within liver and kidney macrophages for 7 days without showing obvious sign of toxicity. The findings from this work provide insights into the diverse biological effects of GF and open new opportunity for their biomedical applications as an interface and scaffold material.

## Supplementary Materials

The following are available online at www.mdpi.com/1996-1944/10/4/336/s1.
