# Supplementary material for: Tracing the Bioavailability of Three-Dimensional Graphene Foam in Biological Tissues
Source: Materials (Basel). 2017 Mar 24;10(4):336. doi: 10.3390/ma10040336 (PMC5506941; doi:10.3390/ma10040336)
Supplement: Supplementary file 1 [file materials-10-00336-s001.pdf]

Supplementary Information for

Tracing the Bioavailability of Three Dimensional Graphene Foam in Biological Tissues

Tanveer A. Tabish[1], Sakineh Chabi[2], Muhammad Ali[3], Yongde Xia[1], Farhat Jabeen[3], and Shaowei Zhang [1,*]

1 College of Engineering, Mathematics and Physical Sciences, University of Exeter, Exeter, EX4 4QF, UK

2 Department of chemistry, Florida Institute of Technology, 150 W. University Blvd Melbourne, Florida 3290, United States

3 Department of Zoology, Government College University, Faisalabad,3800, Pakistan

*Correspondance: s.zhang@exeter.ac.uk, Tel.: +44-1392725276

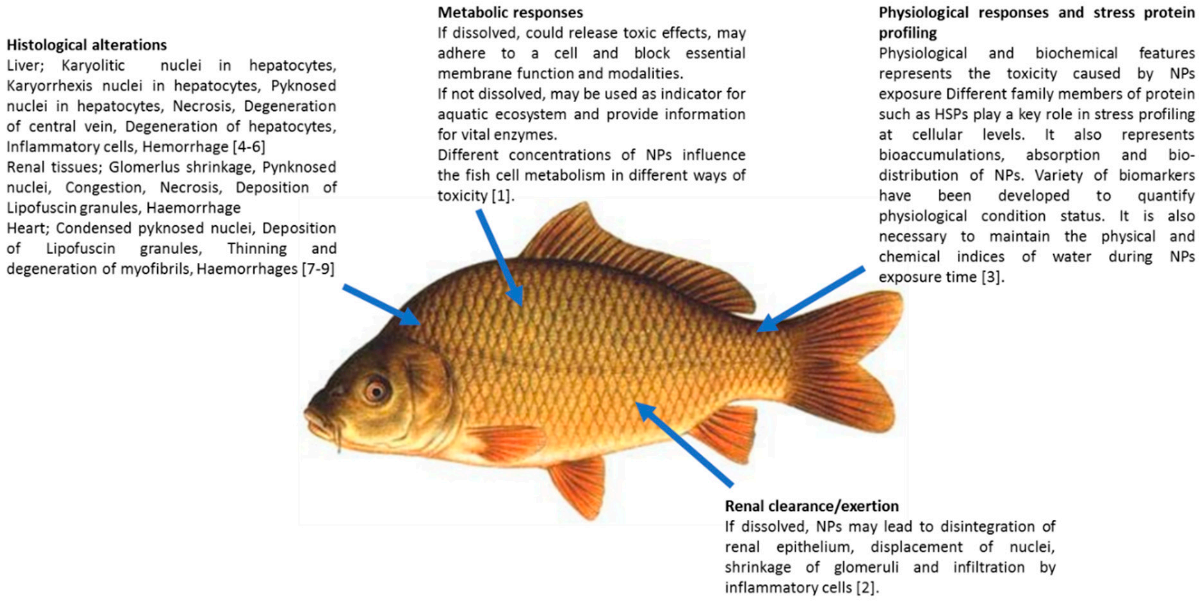

Figure S1. Schematic diagram showing toxicology mechanism of NPs in fish.

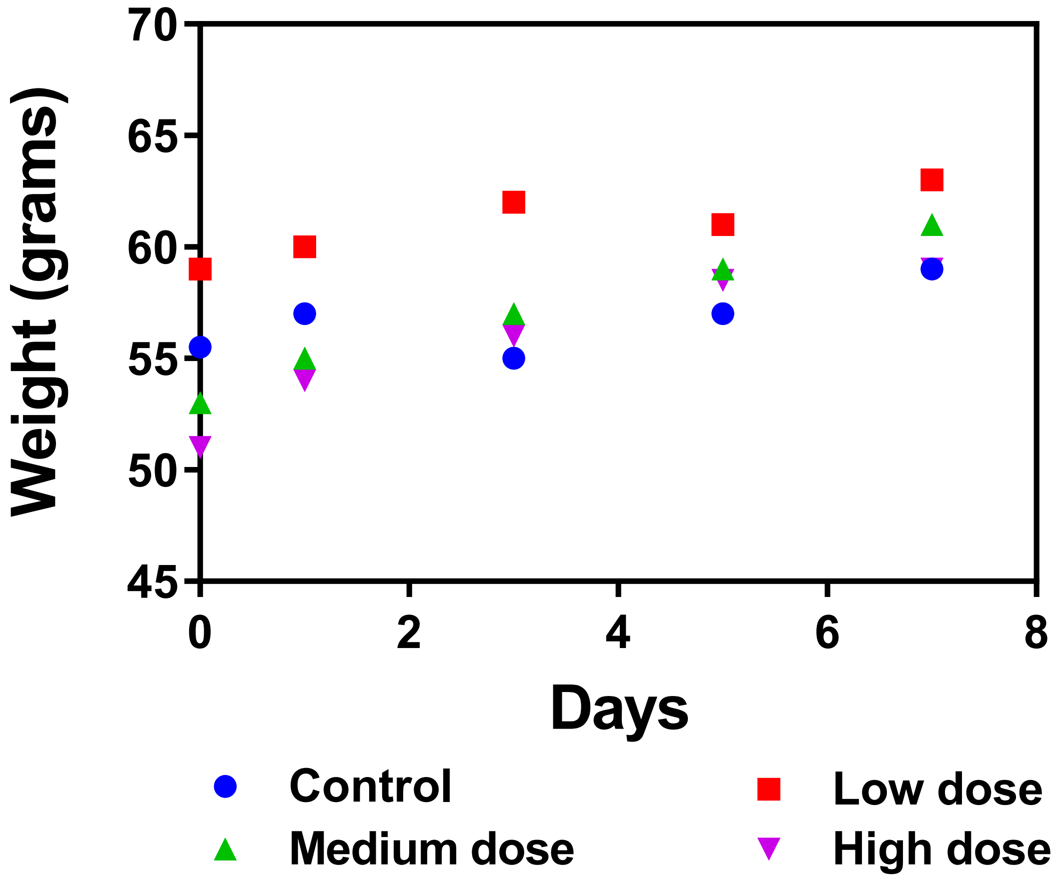

Figure S2. Mean and standard deviations of body weight of common carp treated with GF show no statistically substantial changes over a period of 7 days. Data are presented as mean ± SE (n = 3).

Table S1. Histological findings in fish after treatment with GF for 7 days. Histological changes were observed in both control and treated groups and indicated by + and – (where + means this is found in particular tissue, ++ means this is highly noted in particular tissue, and – means it is not found in the tissue).

Histological alteration	Control	Low dose	Medium dose	High dose
**Liver**				
Karyolitic nuclei in hepatocytes	-	-	+	++
Karyorrhexis nuclei in hepatocytes	-	-	+	++
Pyknosed nuclei in hepatocytes	-<br>-	-<br>-	+	++
Necrosis	-<br>-	-<br>-	+	++
Degeneration of central vein	-	-	+	++
Degeneration of hepatocytes	-	-	+	++
Inflammatory cells	-	-	+	++
Hemorrhage	-	-	-	++
**Renal tissue**				
Glomerlus shrinkage	-	-	+	++
Pynknosed nuclei	-	-	+	++
Congestion	-	-	+	++
Necrosis	-	-	+	+
Deposition of Lipofuscin granules	-	-	+	-
Haemorrhage	-	-	-	++
**Heart**				
Condensed pyknosed nuclei	-	-	-	-
Deposition of Lipofuscin granules	-	-	+	-
Thinning and degeneration of myofibrils	-	-	-	+
Haemorrhages	-	-	-	-

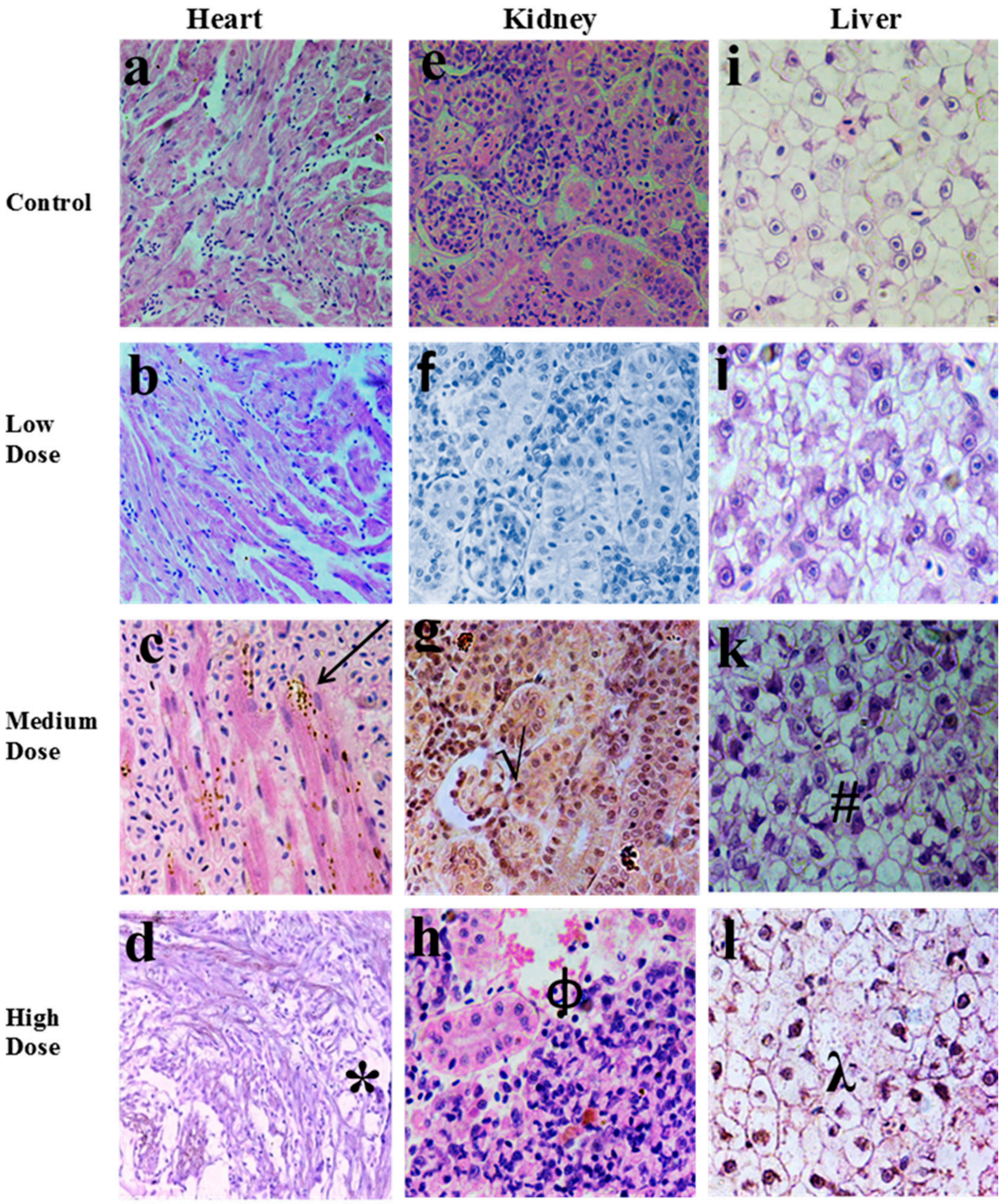

Figure S3. H & E stained light micrographs of *Cyprinus carpio* {heart (a-d), kidney (e-h) and liver tissues (i-l)} treated with GF in dose dependant manner after 5 days. Figures a-b, e-f and i-j show normal histology of heart, kidney and liver. Figures c-d, g-h and k-l show histological alterations in dose dependent manner in selected tissues. Details of histological alterations are given in Table S2. All the images were taken at 50μm scale bar.

Table S2 Histological findings in fish after treatment with GF for 5 days. Histological changes were observed in both control and treated groups and indicated by + and – (where + means this is found in particular tissue, ++ means this is highly noted in particular tissue, and – means it is not found in the tissue).

**Histological alteration**	**Control**	**Low dose**	**Medium dose**	**High dose**
**Liver**				
Karyolitic nuclei in hepatocytes	-	-	+	+
Karyorrhexis nuclei in hepatocytes	-	-	+	++
Pyknosed nuclei in hepatocytes	-<br>-	-<br>-	+	+
Necrosis	-<br>-	-<br>-	+	++
Degeneration of central vein	-	-	+	+
Degeneration of hepatocytes	-	-	+	++
Inflammatory cells	-	-	+	+
Hemorrhage	-	-	-	++
**Renal tissue**				
Glomerlus shrinkage	-	-	+	+
Pynknosed nuclei	-	-	+	++
Congestion	-	-	+	+
Necrosis	-	-	+	+
Deposition of Lipofuscin granules	-	-	+	-
Haemorrhage	-	-	-	++
**Heart**				
Condensed pyknosed nuclei	-	-	-	-
Deposition of Lipofuscin granules	-	-	+	-
Thinning and degeneration of myofibrils	-	-	-	+
Haemorrhages	-	-	-	-

References

1. Sieroslawska, A. & Rymuszka, A. Effects of cylindrospermopsin on a common carp leucocyte cell line. *J. Appl. Toxicol.* **35,** 83-89 (2015).
2. Morgan. J. E. & Morgan. A. J. The accumulation of metals (Cd, Cu, Pb, Zn and Ca) by two ecologically contrasting earthworm species (Lumbricus rubellus and Aporrectodea caliginosa): implications for ecotoxicological testing. *Appl Soil Ecol. 13*(1), 9-20 (1999).
3. Li, Z. H., Zhong, L. Q., Mu, W. N. & Wu, Y. H. Toxicity of Tributyltin in Juvenile Common Carp (Cyprinus Carpio): Physiological Responses, Hepatic Gene Expression, and Stress Protein Profiling. *J Biochem. Mol. Toxic***. 30**, 91-96 (2016).
4. Nowack, B. The behavior and effects of nanoparticles in the environment. *Environ Pollut.* **157,** 1063-1064 (2009).
5. Stoyanova, S., Yancheva, V. S., Velcheva, I., Uchikova, E. & Georgieva, E. Histological Alterations in Common Carp (Cyprinus carpio Linnaeus, 1758) Gills as Potential Biomarkers for Fungicide Contamination. *Braz Biol Techn.* **58**, 757-764 (2015).
6. Handy, R. D., Cornelis, G., Fernandes, T., Tsyusko, O., Decho, A., Sabo-Attwood, T. & Horne, N. Ecotoxicity test methods for engineered nanomaterials: practical experiences and recommendations from the bench. *Environ. Toxicol. Chem*. **31,** 15-31 (2012).
7. Klaine, S. J., Alvarez, P. J., Batley, G. E., Fernandes, T. F., Handy, R. D., Lyon, D. Y. & Lead, J. R. Nanomaterials in the environment: behavior, fate, bioavailability, and effects. *Environ. Toxicol. Chem*. **27,** 1825-1851 (2008).
8. Fabrega, J., Luoma, S. N., Tyler, C. R., Galloway, T. S. & Lead, J. R. Silver nanoparticles: behaviour and effects in the aquatic environment. *Environ Int.* **37,** 517-531 (2011).
9. Choi, H. S. *et* al. Design considerations for tumour-targeted nanoparticles. *Nat. Nanotechnol.* **5,** 42-47 (2010).
